# Prospective open-label trial of personalised connectivity-guided transcranial magnetic stimulation therapy for migraine

**DOI:** 10.1186/s10194-026-02273-7

**Published:** 2026-01-21

**Authors:** Zhen Zhen, Yunze Li, Jie Zhou, Han Li, Andrew Zalesky, Robin Cash, Zhiying Feng, Xianwei Che

**Affiliations:** 1 Centre for Cognition and Brain Disorders, The Affiliated Hospital of Hangzhou Normal University, Building 19, 2318 Yuhangtang Road, Hangzhou, 311121 China; 2 Department of Pain Medicine, First Affiliated Hospital, Zhejiang University School of Medicine, Building 10, 27 Qingchun Road, Hangzhou, 310003 China; 3 Department of Biomedical Engineering, The University of Melbourne, Grattan Street, Parkville, VIC 3010 Australia

**Keywords:** Personalised TMS, Migraine, RSFC, DLPFC, PGC, ROC

## Abstract

**Background:**

Migraine is a leading cause of disability worldwide, affecting hundreds of millions of individuals. Repetitive transcranial magnetic stimulation (rTMS) offers a non-invasive therapeutic option, yet long-term effects remain unclear. We investigated whether rTMS treatment effects could be extended by personalising stimulation targets based on connectivity between the pregenual cingulate cortex (PGC) and dorsolateral prefrontal cortex (DLPFC)-a neural pathway implicated in migraine pathophysiology.

**Findings:**

Twenty-one patients completed all treatments and assessments. Clinical data were analysed using repeated-measures one-way ANOVAs (Baseline, Post, Follow-up), with comparisons being and Bonferroni correction (2-tailed). Personalised rTMS had a large effect on headache frequency (estimated difference, 4.42, *p* = 0.004, 95% CI, 1.30–7.54, Cohen’s d, 0.81) and intensity (estimated difference, 2.54, *p* < 0.001, 95% CI, 0.97–4.10, Cohen d = 0.92), at Follow-up. After treatment, 11/21 (52.38%) patients were classified as responders. The response rate was maintained at 52.38% at Follow-up. Changes in DLPFC-PGC connectivity were able to classify responders from non-responders (AUC, 0.80, *p* = 0.020, sensitivity, 72.70%, specificity, 80%).

**Conclusions:**

In this study, personalised rTMS treatment demonstrated a large and long-term effect for migraineurs. These novel findings need to be directly compared with conventional group-average targeting strategies in future controlled trials.

**Trial registration:**

Chinese Clinical Trials Registry (ChiCTR2400094055).

**Supplementary Information:**

The online version contains supplementary material available at 10.1186/s10194-026-02273-7.

## Introduction

Migraine​​ is characterised by recurrent moderate-to-severe headaches, affecting 1 billion people globally and ranks as the second leading cause of disability [1, 2]. In addition to medications, ​​repetitive transcranial magnetic stimulation (rTMS)​​ has emerged as a safe and non-invasive form of treatment option for migraine. By targeting the ​​dorsolateral prefrontal cortex (DLPFC)​​, rTMS shows promise in reducing migraine frequency and intensity [3–6]. However, our recent meta-analysis revealed ​​unclear long-term effects, with only 3 studies and a synthesised effect size of 0.51 within 8–12 weeks follow-up [7]. These findings raised concerns for the effectiveness of rTMS in migraine management.

Recent evidence suggests that the therapeutic efficacy of rTMS may be enhanced by tailoring stimulation targets to optimally engage disorder-relevant brain circuits [8–10]. In depression, for example, clinical outcomes are improved when rTMS is delivered to regions of the DLPFC that are functionally connected to the subgenual cingulate cortex (SGC), a region implicated in mood regulation [11–13]. Traditional targeting approaches, such as the “5-cm rule” and the Beam F3 method, have some limitations. The former may lead to targeting errors due to individual anatomical variability [14], while the latter approximates the MRI-guided DLPFC location in most participants but still shows an average deviation of several millimetres [15]. Meanwhile, personalised stimulation guided by individual functional connectivity may overcome these issues [11, 16].

In the context of migraine, several lines of evidence indicate a role of the pregenual cingulate cortex (PGC), as well as its connectivity with the DLPFC, suggesting a potential target for neuromodulation. For example, a few studies have revealed abnormal DLPFC–PGC connectivity in individuals with migraine, such as hypoconnectivity in one study [17] and hyperconnectivity in another [18]. This circuity is deemed as abnormal although a consistent hypo- or hyperconnectivity pattern was not evidenced due to variabilities in connectivity methods and patients’ demographics. More importantly, one pioneering study found that a course of DLPFC-rTMS aimed at decrease headache was associated with RSFC between the DLPFC and PGC (BA32) [19]. Moreover, a serotonin reuptake inhibitor selectively increased PGC activity, but not other parts of ACC in migraineurs [20]. Altered serotonergic neurotransmission has long been implicated in the pathophysiology of migraine [21–23]. Overall, these findings suggest that the DLPFC-PGC circuitry could be used to personalise rTMS targeting for migraine management.

The current study employed a prospective design to investigate the efficacy of DLPFC-PGC connectivity guided personalised rTMS treatment for migraine. A course of 10 treatment sessions was delivered to migraineurs (*n* = 21) guided by DLPFC-PGC connectivity over 2 weeks. The association between DLPFC-PGC connectivity and migraine treatment outcome was also examined. It is hypothesised that personalised rTMS treatment would increase long-term effects for migraine, assessed with headache frequency and intensity, compared to a small-to-medium effect size in the literature (0.51). We also hypothesised that DLPFC-PGC connectivity was associated with treatment response for rTMS.

## Methods

### Study overview

**Fig. 1:**
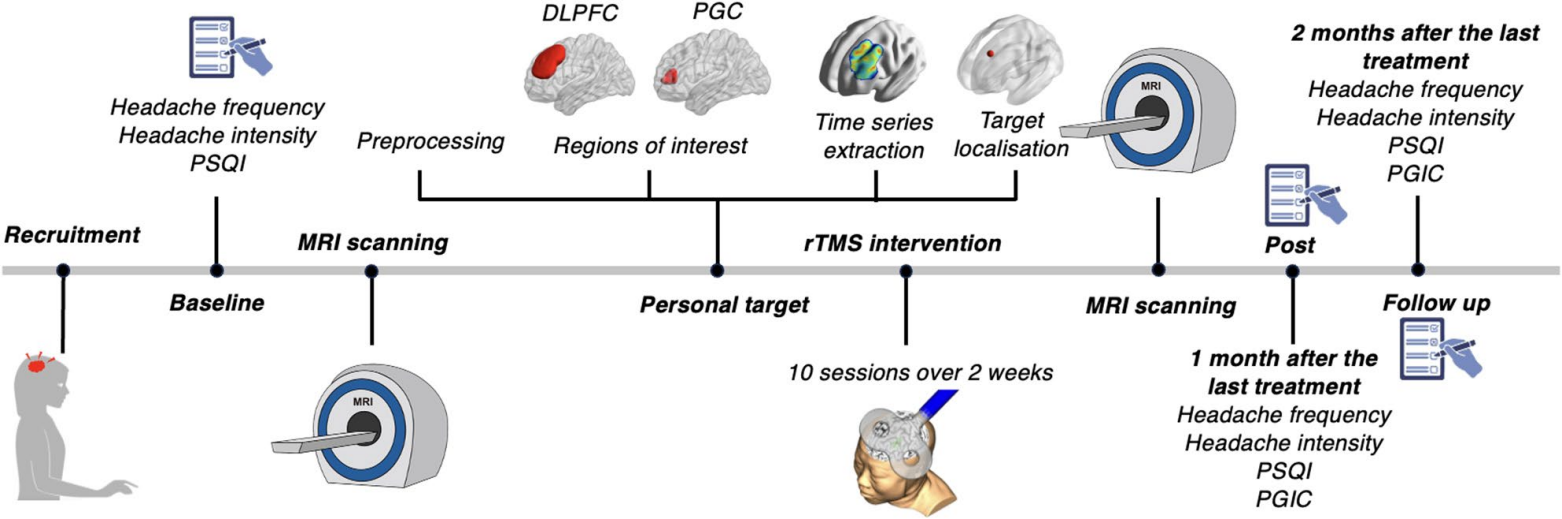
Study design and procedure

We registered the trial in the Chinese Clinical Trials Registry (ChiCTR2400094055) http://www.chictr.org.cn (see Fig. 1). Over two weeks, patients received 10 sessions of 10-Hz rTMS (≥ 24-hour interval). rTMS treatments were performed by Z.Z. who did not participate in clinical assessment. Clinical assessments were performed before the intervention (‘Baseline’), 1 month since the last treatment (‘Post’), and 2 months since the last treatment (‘Follow-up’). All clinical assessments were performed by a trained neurologist (YL). Structural and functional MRI scans were performed both before and after the full course of treatment. The baseline clinical assessment and baseline MRI were conducted within one week of each other, and the post-treatment MRI scan was performed immediately after the final session. All participants voluntarily participated in this study and signed an informed consent form. Ethical approval was obtained from the Ethics Committee at the Affiliated Hospital of Hangzhou Normal University (2024E2HS019). This study was conducted in accordance with the Code of Ethics of the World Medical Association (Declaration of Helsinki).

Individuals with migraine first completed baseline clinical assessments, including headache frequency, intensity, and the Pittsburgh Sleep Quality Index (PSQI). Structural and functional magnetic resonance imaging (MRI) scans were then acquired to generate connectivity maps based on predefined regions of interest (DLPFC and PGC) for individualised target calculation. The optimal stimulation site was determined from these maps. The baseline clinical assessment and baseline MRI were conducted within one week, and the post-treatment MRI scan was performed immediately after the final treatment session. Participants subsequently rTMS targeting the individualised DLPFC site, followed by an immediate post-treatment MRI scan. One- and two-month follow-ups recorded headache frequency, intensity, as well as PSQI and the Patient Global Impression of Change (PGIC).

### Patients

Between December 2024 and March 2025, we recruited 35 patients with migraine. The inclusion criteria were: (1) 18–65 years old; (2) diagnosed with migraine according to the International Classification of Headache Disorders (ICHD-III) [24]; (3) first onset before the age of 50 and a history of migraine > 1 year; (4) attacks ≥ 4 days per month in the 3 months before and during the screening period; (5) signed an informed consent and cooperated with the assessment and rTMS treatment. The exclusion criteria included: (1) TMS contraindications such as metal implants and pacemakers [25]; (2) severe anxiety or depression (17 Hamilton depression items ≥ 35, 14 Hamilton anxiety items ≥ 29); (3) history of adjustment of migraine preventive medication; (4) aphasia or cognitive dysfunction (Mini-Mental State Examination ≤ 23); (5) pregnancy or lactation; (6) severe comorbidities such as tumour. Concomitant medication was permitted during this trial. Each patient’s medication regimen (drug type and dosage) remained consistent throughout all sessions, with no adjustments being made during the treatment or follow-up phases.

### MRI data acquisition and target localisation

#### MRI data acquisition

MRI data were acquired on a GE Architect 3.0 Tesla scanner at the Affiliated Hospital of Hangzhou Normal University. High-resolution structural images were obtained using a 3D T1-weighted magnetization-prepared rapid gradient echo (MP-RAGE) sequence with the following parameters: field of view (FOV) = 256 mm, slice thickness = 1.0 mm, matrix size = 256 × 256, inversion time (TI) = 1100 ms, flip angle = 7°, and receiver bandwidth = 31.25 kHz. The sequence included 192 slices acquired in a single slab with no overlap. Resting-state functional MRI data (600 frames; 10 min scan duration) were collected using a 2D gradient-echo echo-planar imaging (EPI) sequence with the following parameters: TR = 1000 ms, TE = 30 ms, flip angle = 60°, FOV = 216 mm, slice thickness = 3.0 mm, no slice gap, matrix size = 72 × 72, and 50 axial slices acquired in interleaved order. A multiband (HyperBand) factor of 3 and parallel imaging with ARC were used to accelerate acquisition. To correct for EPI distortions, a field map was acquired using a 2D gradient-echo sequence with TR = 400 ms, TE1 = 4.9 ms, TE2 = 7.4 ms, flip angle = 60°, FOV = 216 mm, matrix size = 64 × 64, slice thickness = 3.0 mm, and 50 slices.

### Imaging preprocessing

For each participant, T1-weighted images were manually adjusted to align with the anterior commissure (AC) before preprocessing [26]. The preprocessing of the acquired fMRI data included the following steps: (1) spatial and gradient distortion correction, (2) head motion correction, (3) intensity normalization, (4) one-step spline resampling of EPI frames into 2 mm isotropic MNI space, and (5) application of the FIX + ICA pipeline for the removal of motion-related and other structured noise components. In addition, further preprocessing steps included high-pass temporal filtering (> 0.01 Hz) and spatial smoothing. Given the relatively small size of subcortical structures of interest [16, 27], images were minimally smoothed using a Gaussian kernel (4 mm full width at half maximum, FWHM) to minimise spatial information loss and reduce spurious signal spread across grey and white matter boundaries. FMRI preprocessing was carried out in FSL version 6.0.0 (FMRIB Software Library, Oxford, UK), in combination with custom scripts implemented in MATLAB R2022b (MathWorks, Natick, MA).

### Regions of interest

The DLPFC was defined by combining four 20-mm radius spherical ROIs centred at commonly used stimulation targets in the left prefrontal cortex [8, 16]. These included coordinates derived from Brodmann areas BA9 (MNI = -36, 39, 43) and BA46 (MNI = -44, 40, 29), as well as standardised TMS targets including the “5-cm rule” site (MNI = -41, 16, 54) [28], as well as the Beam F3 group-average site (MNI = -37, 26, 49). These spheres were constructed separately and then merged to create a comprehensive DLPFC mask. The PGC mask was obtained from an openly available GitHub repositor-y (available at: https://github.com/danbang/article-private-public/tree/master/ROI_Masks) [29], following the mask generation method described by Bang & Fleming [30]. Specifically, this mask corresponds to the pregenual anterior cingulate cortex, defined within the medial prefrontal cortex using the coherence × distance second-level t-map approach, which highlights voxels functionally connected to the default mode network. In our study, this PGC mask was used as the target ROI to assess its functional connectivity with the dorsolateral prefrontal cortex (DLPFC). This mask served as the target ROI to assess connectivity with DLPFC in our analysis.

### Time series extraction

A seedmap approach was used to enhance signal-to-noise ratio [16, 28]. The DLPFC time series were derived by averaging the fMRI signal across all voxels within the predefined DLPFC mask. Specifically, the PGC time series was computed as a weighted spatial average of the fMRl data across all gray matter voxels [28], excluding the DLPFC. Gray matter voxels were weighted according to their group-averaged connectivity with the PGC time series. The single group-average connectivity map, which was used to weight each voxel’s contribution to the PGC time series, was derived from 1200 scans (600 individuals x 2 scans). The data used to generate this group-averaged functional connectivity map were obtained from the HCP dataset [31, 32]. The signal to noise ratio improved because data from most of gray matter was used to estimate the PGC time series, as opposed to the approximately 20 voxels comprising the PGC.

### Target localisation

The optimal stimulation target was defined as the location within the DLPFC showing the strongest functional connectivity with PGC. Within the DLPFC, we identified the top 0.5% of voxels with the strongest connectivity to the PGC. Spatial clustering was then applied, and the centroid of the largest contiguous cluster was selected as the stimulation target [16]. In order to validate the induced electric field, electric field simulations for the D-70 mm Air Film Coil were performed using the SimNIBS modelling environment [33], which utilizes a finite element model of brain current flow based on individual structural images and stimulation intensity.

### rTMS treatment protocol

Migraineurs received 10 sessions of rTMS treatment within two weeks (≥ 24-hour interval). Each session was delivered at 10 Hz with 5-sec trains and 25-sec intervals, for a total of 36 trains and 1800 pulses (18 min) [34]. During treatment, the coil handle was positioned at 180° relative to the sagittal plane and the patients’ treatment targets in MNI space had the following mean coordinates: -30.26 ± 9.25, 34.30 ± 8.87, 37.69 ± 8.50. The stimulation intensity was set at 100% of the resting motor threshold (RMT), which was defined as the minimum stimulation intensity to elicit motor-evoked potentials (MEPs) greater than 0.05 mV in the first dorsal interosseous muscle in at least 5 out of 10 trials. Both RMT and rTMS were delivered by a figure-of-eight coil connected to a Magstim Rapid^2^ system (Magstim Company Ltd, UK). Individual T1 images were reconstructed to create an individual 3D model of the brain. All treatment sessions were performed with a neuronavigation system for precise localisation of the target (Brainsight 2.3; Rogue Research, Canada).

### Outcome measures

The outcome measures were reported according to the IMMPACT recommendations for chronic pain clinical trials [35]. The primary outcome measure was headache days in the last month, as registered in the headache diary [36]. Treatment response was defined as ≥ 30% reduction in the number of headache days [37]. Secondary outcome measures included headache intensity, Pittsburgh sleep quality index [38], patient global impression of change [39], and brain functional connectivity. It is worth noting that patient global impression of change was assessed at Post and Follow-up but not Baseline.

Electric field simulations were performed using SimNIBS 4.0 with the D70 mm figure-8 coil and individualised head models generated from each participant’s structural MRI. The stimulation intensity was set according to each participant’s resting motor threshold (RMT), and the corresponding di/dt values were used as input parameters for the simulations. Quantitative electric field indices were extracted, including the mean electric field strength within the target region, peak electric field amplitude, and the percentage of target voxels exceeding a predefined threshold (threshold set at 150 V/m).

### Statistical analysis

Normality was initially assessed for the outcome measures. Headache frequency was analysed using repeated-measures one-way ANOVAs with three time points (Baseline, Post, and Follow-up). Pairwise comparisons were performed with Bonferroni correction at *p* ≤ 0.05. PGIC was analysed using Chi-square test with three categories (Improvement, No change, Worsening). Receiver operating characteristic (ROC) analysis was performed to classify responders and non-responders with functional connectivity data. All statistical analyses used two-tailed tests. Because headache intensity and PSQI scores did not conform to a normal distribution, the Kruskal–Wallis test was used to evaluate differences across the three time points.

## Results

### Demographic and clinical information of patients

**Fig. 2:**
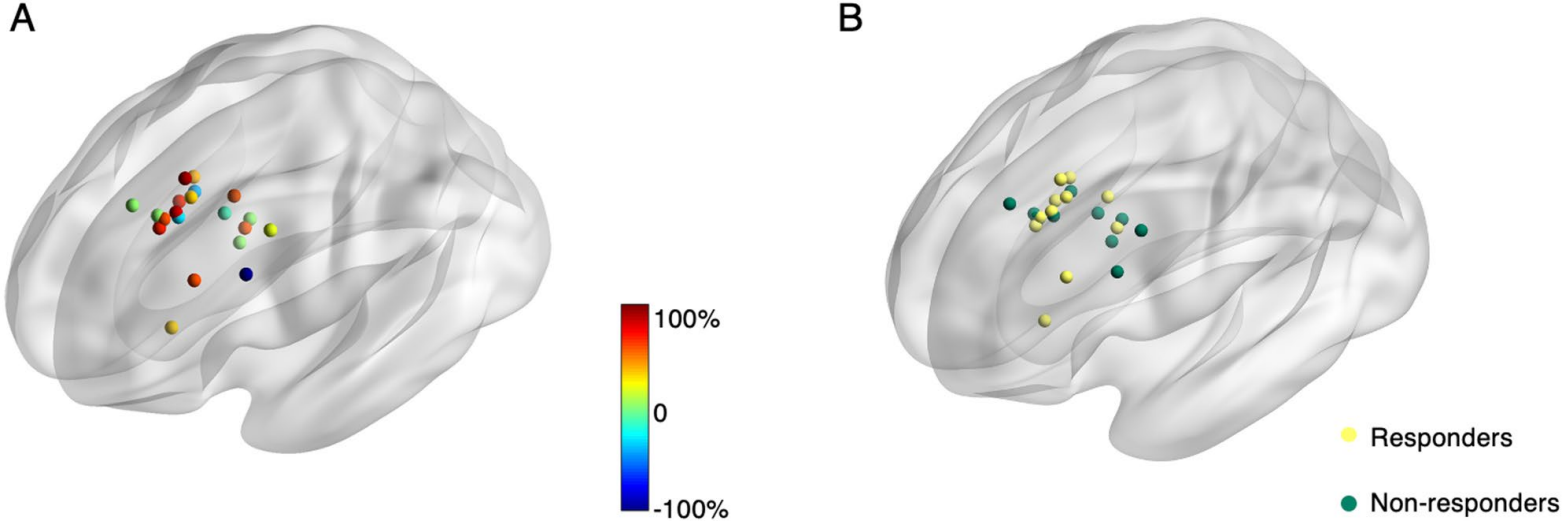
Individual treatment targets. (**A**) Colors indicate percent changes in headache frequency, with warm and hot colors indicating more reduction (**B**) The colours represent responders and non-responders

Of 35 patients screened, 12 declined participations, and 2 were excluded due to baseline headache intensity < 3. Details are presented in the CONSORT flow diagram (Supplement 1). Thus, 21 participants finished the treatment and assessments (Table 1). Most of the patients were female (15/21). Most of the patients reported headache on both hemispheres (14/21). They experienced headache on ~ 10 days per month with a medium level of intensity (5.67/10). Individual treatment targets are presented in Fig. 2. Adverse effects were assessed during the treatment. Two patients reported mild scalp discomfort after the first treatment session, which was alleviated in subsequent sessions. No other adverse effects, such as headache exacerbation, syncope, or seizures, were observed. The mean resting motor threshold (RMT) of the 21 patients was 55 (SD = 8.76).

**Table 1:** Demographic and clinical characteristics of headache patients

Demographic Measure Headache patients	
*N* 21 *Age*,* years* Mean ± SD 36.7 ± 10.4 (20–61) *Gender* Female, n (%) 15 (71.4%) Male, n (%) 6 (28.6%) *Year of education* Mean ± SD 16.0 ± 2.6 (9–19) *Headache location* Right, n (%) 3 (14.3%) Left, n (%) 4 (19.0%) Bilateral 14 (66.7%) *Aura* With aura, n (%) 12 (57.1%) Without aura, n (%) 9 (42.9%) *Headache days (past 1-month before treatment)* Mean ± SD 10.2 ± 7.1 (3–30) *Headache intensity (1-past month before treatment*,* NRS)* Mean ± SD 5.7 ± (3–9) *MIDAS* Median (IQR) 34 (1-310) *Duration of illness* Mean ± SD 12.3 ± 5.9 (1–20) *Medication use* Preventive medication use, n (%) 7 (33.3%) Acute medication use, n (%) 19 (90.5%) Medication regimen stable during study, n (%) 21 (100%)	

Note: Data are presented as mean ± SD or median (IQR), unless otherwise specified.NRS = Numeric Rating Scale; MIDAS = Migraine Disability Assessment Scale.Preventive medications included propranolol, flunarizine, traditional Chinese medicine, and vitamin B2. Acute medications included nonsteroidal anti-inflammatory drugs (NSAIDs; e.g., ibuprofen, loxoprofen, etoricoxib, EVE, flurbiprofen) and triptans

### Primary outcome

**Fig. 3:**
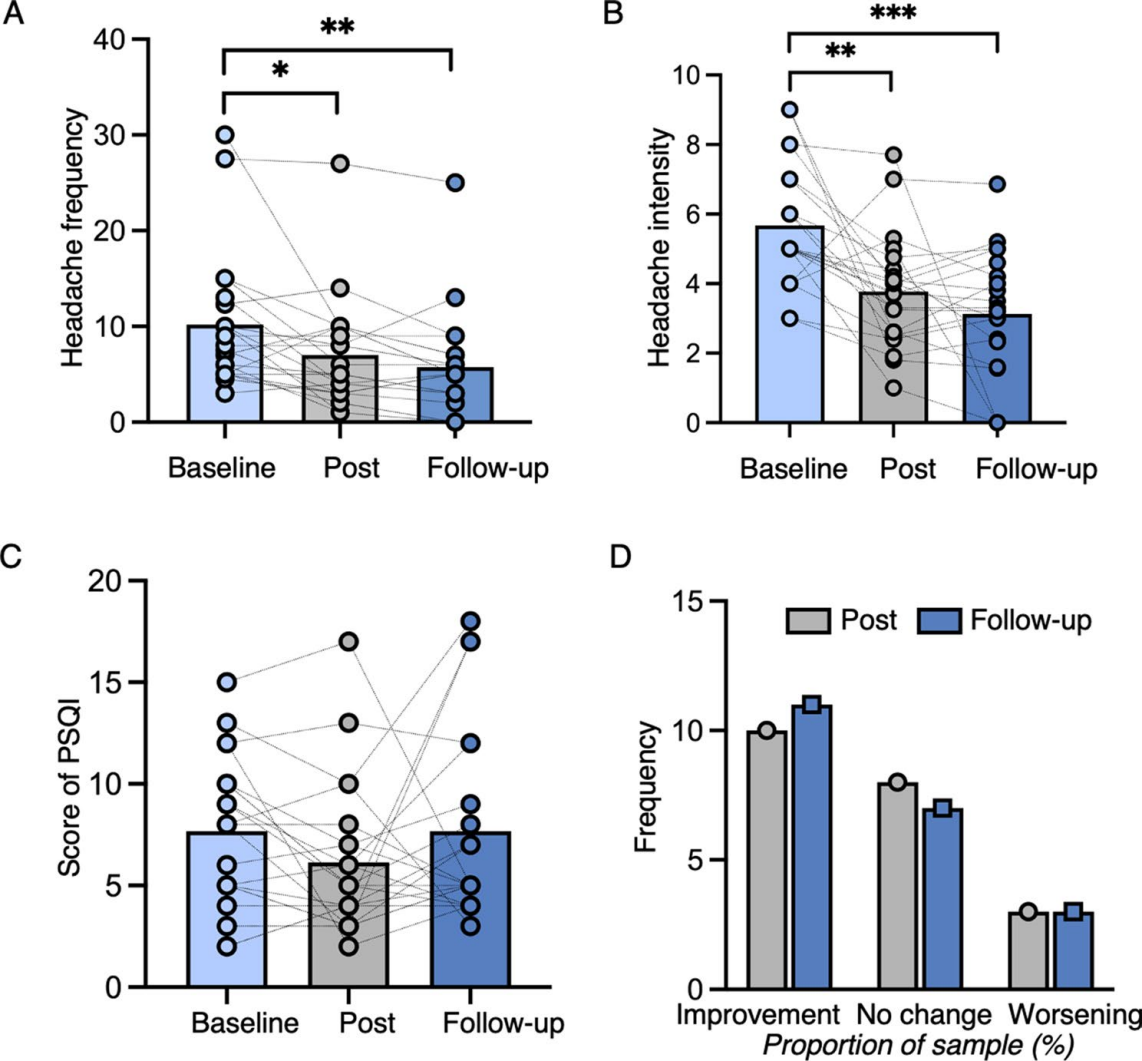
Clinical outcomes. (**A**) Headache days were decreased after rTMS treatment (*p* = 0.044, Cohen’s d = 0.58), and this effect was maintained at 2-month follow-up with a large effect size (*p* = 0.004, Cohen’s d = 0.81). (**B**) The same outcomes were observed for pain intensity (Post: *p* = 0.003, Cohen d = 0.83; Follow-up: *p* = 0.001, Cohen d = 0.92). (**C**) No treatment effect was observed for sleep quality (PSQI) (*p* = 0.308). (**D**) Patient global impression of change (assessed at Post and Follow-up) indicated that half of the patients reported improvement at post (10/21) and follow-up (11/21)

There was a significant time effect on headache frequency (*F*_2, 19_ = 13.66, *p* = 0.001). Headache days were decreased after rTMS treatment (estimated difference, 3.18, *p* = 0.044, 95% CI, 0.07–6.29, Cohen’s d, 0.58), and this effect was maintained at follow-up with a large effect size (estimated difference, 4.42, *p* = 0.004, 95% CI, 1.30–7.54, Cohen’s d, 0.81) (Fig. 3A). At Post, the mean headache days decreased by 3.18 ± 5.46 days (a 31.24% reduction). At the Follow-up, the mean headache days decreased by 4.42 ± 5.48 days (a 43.41% reduction). After treatment, 11/21 (52.38%) patients were classified as responders. The response rate was maintained at 52.38% at Follow-up.

### Secondary outcomes

A time effect was also observed on headache intensity (*H*_2, 19_ = 29.65, *p* < 0.001, η² = 0.461). rTMS treatment decreased headache intensity at Post (*p* < 0.001, *r* = 0.66) and maintained this effect to Follow-up (*p* < 0.001, *r* = 0.78) (Fig. 3B).

There was no effect on sleep disturbance (*H*_2, 19_ = 2.747, *p* = 0.253, η² = 0.012) (Fig. 3C). For patient global impression of change, 10/21 (47.62%) reported improvement, 8 (38.10%) reported no change, and 3 (14.29%) reported worsening at Post. At Follow-up, 11 (52.38%), 7 (33.33%), and 3 (14.29%) patients reported improvement, no change, and worsening respectively (Fig. 3D). At Post, PGIC was negatively correlated with the reduction rate in headache frequency (Spearman’s rho = − 0.492, *p* = 0.023), indicating that self-reported benefit aligns with headache relief.

### ROC results and differences in treatment response

**Fig. 4:**
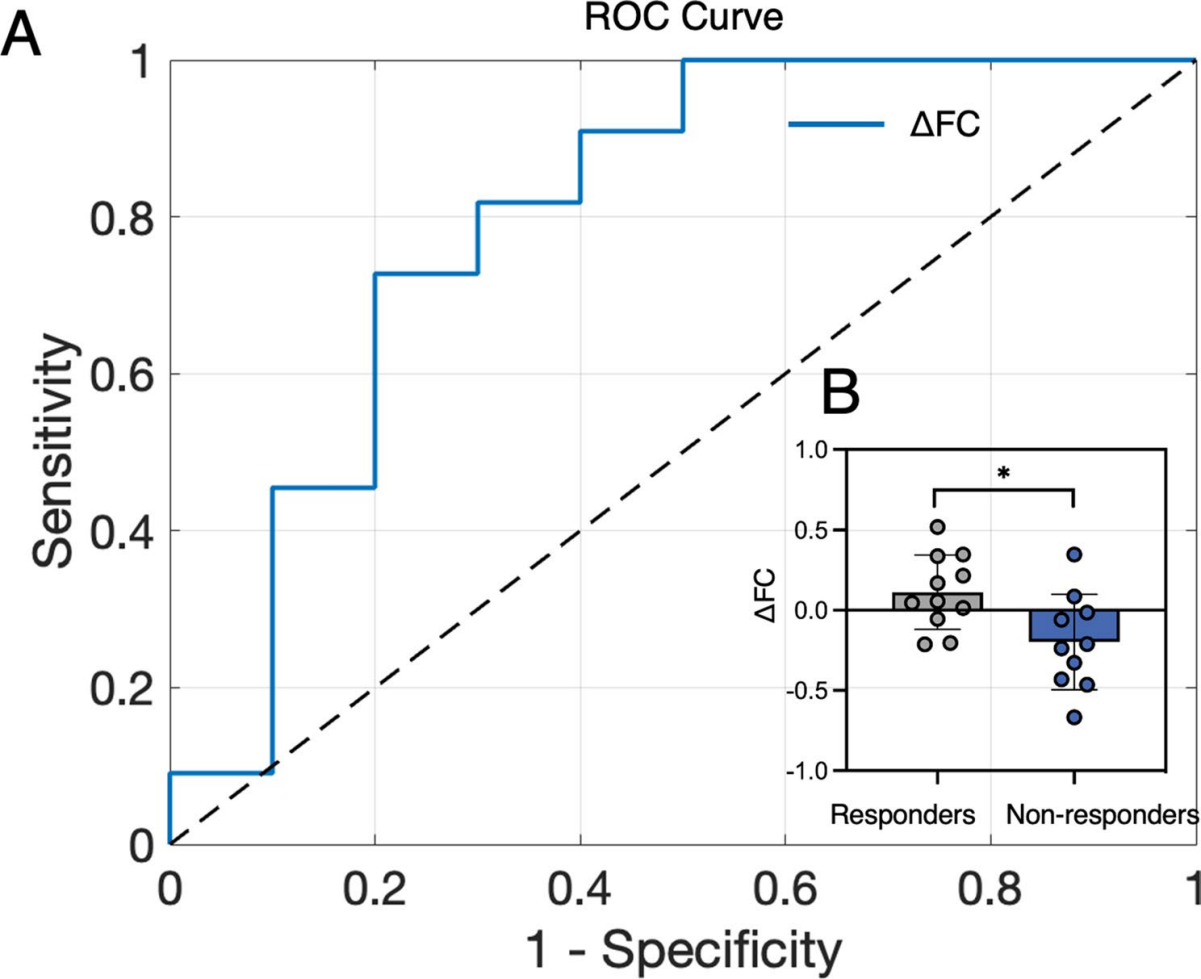
RSFC association with treatment response. (**A**) Changes in DLPFC-PGC functional connectivity were able to classify responders from non-responders at Post (AUC = 0.80, *p* = 0.020). (**B**) Responders showed increased DLPFC-PGC connectivity compared to non-responders (*p* = 0.0148)

Receiver operating characteristic (ROC) analysis revealed that changes in DLPFC-PGC functional connectivity (ΔFC) were able to classify responders from non-responders at Post stage (AUC = 0.80, *p* = 0.020, sensitivity = 72.7%, specificity = 80%) (Fig. 4). These findings suggest that DLPFC-PGC connectivity may serve as a good biomarker for treatment response [40]. Independent sample t-test revealed that responders showed increased DLPFC-PGC connectivity compared to non-responders (t = 2.679, *p* = 0.015). However, DLPFC-PGC functional connectivity did not change significantly after treatment in the overall sample (mean FC _Pre_ = 0.411, mean FC _Post_ = 0.376, *p* = 0.598).

### E-field results

Individualised coordinates of the 21 participants and result of E-field are provided in Supplement 2. Patients showed an average electric field strength of 92.85 ± 35.84 within the ROI, with a maximum electric field strength of 184.77 ± 65.29. The percentage of ROI volume exceeding the threshold was 77.38 ± 28.22. Correlation analyses with clinical outcomes revealed that the percentage of suprathreshold ROI volume was negatively correlated with headache frequency at follow-up (*r* = − 0.516, *p* = 0.017).

## Discussion

This open-label clinical trial, to our knowledge, was the first connectivity-guided personalised rTMS trial for chronic pain. Positive effects of DLPFC-rTMS have been reported in migraineurs [3–6, 41]. However, long-term effects (2–3 months) were modest (0.51 effect size) and inconsistent (3 studies) [4, 6, 42]. With personalised targeting, the current study achieved a large effect of 0.81 on headache frequency at 2-month follow-up. The same effect was observed for headache intensity (0.92). These benefits are numerically better than the synthesised effect in our latest meta-analysis (0.51). It is noted that the meta-analysis used SMD, we thus calculated the effect sizes using this method, which were 0.74 and 1.41, respectively [7]. In addition, our data demonstrated > 50% response rate at 2-month follow-up, whereby the literature generally reported ~ 50% response rate at post-treatment but no such effects at 2-month follow-up [19, 41, 42]. These findings provide prospective evidence for the clinical effectiveness of precision RSFC-personalised rTMS for migraine.

In addition to clinical efficacy, we further provided novel evidence that DLPFC-PGC connectivity was associated with rTMS treatment effect in migraine. Migraineurs who responded better to rTMS treatment showed increased DLPFC-PGC connectivity aftertreatment. These findings indicate that clinical efficacy was associated with parallel changes in neural connectivity metrics. These data support and extend beyond previous findings whereby a course of DLPFC-rTMS treatment increased DLPFC-PGC connectivity in migraine [19]. These findings also support the critical role of PGC in the pathophysiology of migraine, which is sensitive to brain serotonin and contributes to recurring headache attacks [20]. We also performed ROC analysis between baseline functional connectivity and responders. A priori predictor would have substantially greater clinical significance. However, the result was not significant (AUC = 0.282, *p* = 0.091).

The clinical efficacy of RSFC-personalised rTMS could be explained in a few perspectives. This personalised rTMS strategy may works to improve the targeting accuracy for a certain connection. Indeed, our data showed that the volume of the target region reaching an effective electric field correlated with headache relief, offering preliminary support for this network-specific mechanism. It is widely accepted the PGC forms an essential component of the pain matrix [43, 44]. PGC is believed to be involved in pain modulation, in which the emotional responses to pain are modified, such as fear, anxiety, and distress [45–47]. Our data revealed that treatment responders demonstrated higher DLPFC-PGC connectivity compared to the non-responders, supporting a better commination within this circuit and the capacity for pain modulation. In addition, there is a line of evidence on neurotransmission that may be involved in the treatment effect on migraine. Specifical, serotonergic neurotransmission is implicated in the pathophysiology of migraine [21–23], in which a serotonin reuptake inhibitor selectively increased PGC activity [20]. Moreover, the effects of DLPFC-rTMS on pain emotions were found to be modulated by dopaminergic transmissions [45–47]. Overall, these findings help explain a potential superior efficacy of RSFC-personalised rTMS for migraine.

Previous studies have shown that resting-state alterations of prefrontal–cingulate/midbrain pathways in migraine are heterogeneous, but they commonly involve abnormal activation or coupling within the prefrontal–descending pain modulation system [48]. From a neurobiological perspective, the DLPFC, as a higher-order cognitive and emotional regulatory region, can engage in descending pain modulation via the cingulate cortices and midbrain structures such as the periaqueductal grey (PAG) [49]. In line with this argument, treatment responders demonstrated higher DLPFC-PGC connectivity in our data. This interpretation is consistent with previous studies showing that, following pain relief, functional or structural abnormalities in prefrontal and cingulate networks can partially revert toward levels observed in healthy individuals [46, 50]. However, the current study did not provide data to demonstrate a hypo- or hyperconnectivity pattern compared to healthy controls. This calls for future studies to design baseline controls for migraine rTMS trials.

Conventional group-average targets such as the 5 cm rule or Beam F3 ignore individual differences in DLPFC anatomy and its connectivity with deeper nodes. The induced electric field may miss the circuits that drive the treatment effects in some patients. Functional connectivity–guided personalisation selects the DLPFC target with the strongest coupling to the PGC from each patient’s individual connectivity map to avoid this mismatch. This strategy could maximise network coverage and therefore has a greater chance of producing durable, robust clinical effects.

We also assessed the effects on patient impression of change. Just over half of the patients (11/21) reported positive changes at 2-month follow-up. Overall, the patient impression of treatment was consistent with the results of headache frequency at both post (47.62%, 52.38%) and follow-up (52.38%, 52.38%). Moreover, self-reported benefit by PGIC was associated with headache relief. These findings highlight the consistency of treatment effects. Our short-term follow-up data (1-month) also largely replicated the medium-to-large effect in the synthesis (0.8 effect size at 1–4 weeks) [7]. Our participants exhibited relatively high and broadly distributed electric field intensity within the regions of interest (ROIs). The proportion of ROI volume exceeding the threshold was negatively correlated with headache frequency at follow-up. This finding suggests that the volume of the ROI reaching an effective stimulation intensity may be an important factor influencing clinical efficacy.

It is noted that sleep quality was not improved by the treatment. It is likely that our patients had only very mild sleep disturbance before treatment (Mean = 7.67) [38, 51]. Current evidence shows that sleep disturbances are more prevalent among individuals with migraine [52]. Patients with chronic migraine and/or medication overuse display significantly higher rates of insomnia and sleep fragmentation than those with episodic migraine [53]. TMS studies in migraine patients with comorbid depression or anxiety have reported concurrent improvements in sleep [54], presumably because neuromodulation of mood/arousal networks indirectly alleviates sleep disturbances and, in turn, yields additional benefits for headache symptoms. Future work could stratify treatment strategies by migraine subtype to deliver more targeted care.

There were some limitations in this study, mainly including an open-label design. This is considered a proof-of-concept or feasibility study before embarking on more resource-intensive controlled trials. Our findings need to be directly compared in future controlled trials with conventional group-average targeting strategies. For instance, a head-to-head comparison between personalised and group-average targeting treatment would be able to reveal the superiority of a personalised treatment. Although our sample size is relatively small, it exceeds that in most active arms in migraine rTMS trials [7]. Moreover, the absence of cross-validation or an independent validation cohort introduces a potential risk of overfitting in the ROC results. Future works are needed for external validation. We also acknowledge the short follow-up period. It is noted that migraine is predominantly bilateral in many patients (14/21), with the treatment being performed only in the left DLPFC. This was designed as our previous meta-analysis identified all (8/8) DLPFC migraine trials targeting the left hemisphere [7].

Given these limitations, our novel findings still provide translational prospects for the precision RSFC-personalised rTMS treatment for migraine. However, from a clinical translational perspective, RSFC-guided individualised target localisation entails technical and resource-related barriers, including the acquisition of high-quality resting-state fMRI data, robust analytical pipelines, as well as a neuronavitagion system with costs. These requirements may limit the immediate scalability of this approach in routine clinical settings. Future multicentre studies with larger samples are needed to further assess the reproducibility and practical feasibility of this strategy.

To conclude, this is the first study to investigate personalised connectivity based TMS in migraine, or more broadly, in chronic pain. This personalised therapeutic approach achieved a large effect size in terms of headache frequency and intensity, extending to 2-month follow-up. Association between clinical response and connectivity changes provides support for the anticipated mechanism of this personalised connectivity-based treatment strategy. Overall, the present study presents promising findings that warrant future follow-up in double-blind randomised controlled studies.

## Supplementary Information

Below is the link to the electronic supplementary material.

Supplementary Material 1

Supplementary Material 2

## Data Availability

All data generated or analysed during this study are included in this published article.
