# Supplementary material for: Prospective open-label trial of personalised connectivity-guided transcranial magnetic stimulation therapy for migraine
Source: J Headache Pain. 2026 Jan 21;27(1):44. doi: 10.1186/s10194-026-02273-7 (PMC12905967; doi:10.1186/s10194-026-02273-7)
Supplement: Supplementary file 1 — Supplementary Material 1 [file 10194_2026_2273_MOESM1_ESM.doc]

**Modified CONSORT flow diagram for individual randomized controlled trials of nonpharmacologic treatments.**

An extra box per intervention group relating to care providers and centers has been added.

IQR = interquartile range; max = maximum; min = minimum

**Enrollment**

**Patients**

**Allocation :**

**Patients**

**Follow-up**

**Patients**

**Analysis**

**Patients**

**Allocation:**

**Care Providers**

Assessed for eligibility

(n = 35)

Allocated to treatment(n = 21)

Received allocated treatment (n = 21)

Did not receive allocated treatment (n = 0)

Excluded (n = 14)

Refused to participate (n =12)

Were excluded due to baseline headache intensity <3 (n = 2)

Lost to follow-up (give reason) (n = 0)

Discontinued intervention (give reason) (n = 0)

Analysed (n = 21)

Excluded from analysis (n = 0)

Care providers (n =1), teams (n = 3), centers (n= 3) performing the intervention

Number of patients treated by this care provider (21)
