# Supplementary material for: Prospective open-label trial of personalised connectivity-guided transcranial magnetic stimulation therapy for migraine
Source: J Headache Pain. 2026 Jan 21;27(1):44. doi: 10.1186/s10194-026-02273-7 (PMC12905967; doi:10.1186/s10194-026-02273-7)
Supplement: Supplementary file 2 — Supplementary Material 2 [file 10194_2026_2273_MOESM2_ESM.docx]

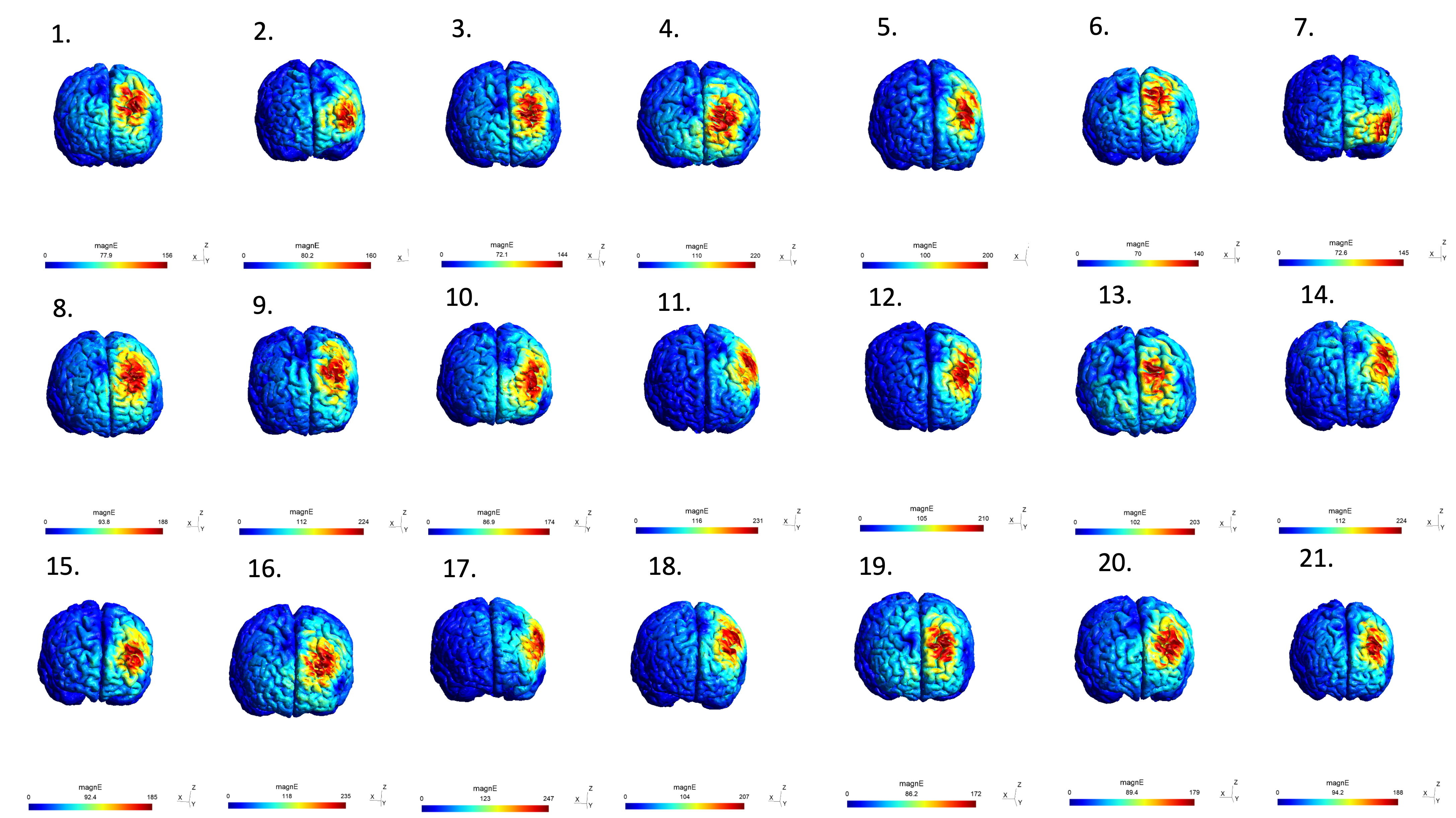


eFig. 1 **Individualized connectivity-guided stimulation targets across participants.**

Resting-state functional connectivity maps between the left dorsolateral prefrontal cortex (DLPFC) and the pregenual anterior cingulate cortex (PGC) were generated for each of the 21 patients with migraine. Warmer colors (red-yellow) indicate stronger positive functional connectivity, and cooler colors (blue) indicate weaker connectivity. The peak connectivity location within the left DLPFC was identified for each participant and used as the individualized rTMS stimulation target. The mean electric field intensity in the patients’ DLPFC was significantly negatively correlated with baseline headache intensity (r = -0.505, p = 0.02).

| x | y | z | MNI coordinate (x,y,z) | 100%RMT |
| --- | --- | --- | --- | --- |
| -21.94 | 28.88 | 40.36 | -23.60, 39.36, 43.44 | 46 |
| -38.41 | 38.46 | 12.79 | -37.67, 46.60, 11.81 | 42 |
| -23.16 | 25.64 | 51.24 | -24.94, 41.75, 42.93 | 40 |
| -18.12 | 19.67 | 43.45 | -19.57, 35.40, 44.13 | 67 |
| -19.89 | 25.8 | 42.49 | -26.22, 28.70, 41.60 | 55 |
| -24.52 | 29.37 | 41.72 | -23.58, 37.29, 42.74 | 43 |
| -42.30 | 32.99 | 28.36 | -43.42, 38.20, 24.11 | 48 |
| -22.85 | 23.53 | 44.57 | -26.26, 32.29, 45.40 | 57 |
| -18.65 | 20.47 | 48.69 | -21.96, 28.78, 47.89 | 66 |
| -43.53 | 16.36 | 26.94 | -47.07, 29.40, 25.47 | 51 |
| -37.63 | 13.54 | 38.66 | -41.58, 16.92, 38.98 | 60 |
| -30.33 | 13.16 | 45.77 | -29.64, 24.45, 45.46 | 56 |
| -21.68 | 31.87 | 39.03 | -25.87, 38.19, 38.58 | 67 |
| -32.01 | 17.28 | 41.91 | -31.69, 18.66, 37.67 | 60 |
| -19.9 | 32.03 | 36.93 | -23.19, 41.83, 34.15 | 51 |
| -20.15 | 33.16 | 39.79 | -19.43, 43.16, 41.22 | 70 |
| -42.66 | 7.81 | 38.78 | -45.38, 19.54, 39.26 | 65 |
| -36.66 | 11.36 | 40.64 | -40.27, 21.22, 41.27 | 53 |
| -17.46 | 40.1 | 43.52 | -23.32, 47.06, 36.92 | 53 |
| -21.79 | 33.46 | 36.52 | -24.95, 43.18, 34.26 | 56 |
| -38.83 | 15.92 | 35.05 | -41.60, 28.81, 33.34 | 49 |
